# Bayesian workflow for the investigation of hierarchical classification models from tau-PET and structural MRI data across the Alzheimer’s disease spectrum

**DOI:** 10.3389/fnagi.2023.1225816

**Published:** 2023-10-18

**Authors:** Clyde J. Belasso, Zhengchen Cai, Gleb Bezgin, Tharick Pascoal, Jenna Stevenson, Nesrine Rahmouni, Cécile Tissot, Firoza Lussier, Pedro Rosa-Neto, Jean-Paul Soucy, Hassan Rivaz, Habib Benali

**Affiliations:** ^1^Department of Electrical and Computer Engineering, Concordia University, Montréal, QC, Canada; ^2^PERFORM Centre, Concordia University, Montréal, QC, Canada; ^3^The Neuro (Montreal Neurological Institute-Hospital), McGill University, Montréal, QC, Canada; ^4^Department of Neurology and Neurosurgery, McGill University, Montréal, QC, Canada; ^5^Translational Neuroimaging Laboratory, McGill University Research Centre for Studies in Aging, Alzheimer’s Disease Research Unit, Douglas Research Institute, Le Centre intégré universitaire de santé et de services sociaux (CIUSSS) de l’Ouest-de-l’Île-de-Montréal, and Departments of Neurology, Neurosurgery, Psychiatry, Pharmacology and Therapeutics, McGill University, Montréal, QC, Canada; ^6^McConnell Brain Imaging Centre (BIC), Montreal Neurological Institute, McGill University, Montréal, QC, Canada

**Keywords:** Alzheimer’s disease, Bayesian workflow, classification, hierarchical modeling, tau-positron emission tomography (PET), magnetic resonance imaging (MRI)

## Abstract

**Background:**

Alzheimer’s disease (AD) diagnosis in its early stages remains difficult with current diagnostic approaches. Though tau neurofibrillary tangles (NFTs) generally follow the stereotypical pattern described by the Braak staging scheme, the network degeneration hypothesis (NDH) has suggested that NFTs spread selectively along functional networks of the brain. To evaluate this, we implemented a Bayesian workflow to develop hierarchical multinomial logistic regression models with increasing levels of complexity of the brain from tau-PET and structural MRI data to investigate whether it is beneficial to incorporate network-level information into an ROI-based predictive model for the presence/absence of AD.

**Methods:**

This study included data from the Translational Biomarkers in Aging and Dementia (TRIAD) longitudinal cohort from McGill University’s Research Centre for Studies in Aging (MCSA). Baseline and 1 year follow-up structural MRI and [^18^F]MK-6240 tau-PET scans were acquired for 72 cognitive normal (CN), 23 mild cognitive impairment (MCI), and 18 Alzheimer’s disease dementia subjects. We constructed the four following hierarchical Bayesian models in order of increasing complexity: (Model 1) a complete-pooling model with observations, (Model 2) a partial-pooling model with observations clustered within ROIs, (Model 3) a partial-pooling model with observations clustered within functional networks, and (Model 4) a partial-pooling model with observations clustered within ROIs that are also clustered within functional brain networks. We then investigated which of the models had better predictive performance given tau-PET or structural MRI data as an input, in the form of a relative annualized rate of change.

**Results:**

The Bayesian leave-one-out cross-validation (LOO-CV) estimate of the expected log pointwise predictive density (ELPD) results indicated that models 3 and 4 were substantially better than other models for both tau-PET and structural MRI inputs. For tau-PET data, model 3 was slightly better than 4 with an absolute difference in ELPD of 3.10 ± 1.30. For structural MRI data, model 4 was considerably better than other models with an absolute difference in ELPD of 29.83 ± 7.55 relative to model 3, the second-best model.

**Conclusion:**

Our results suggest that representing the data generating process in terms of a hierarchical model that encompasses both ROI-level and network-level heterogeneity leads to better predictive ability for both tau-PET and structural MRI inputs over all other model iterations.

## 1. Introduction

Alzheimer’s disease (AD) dementia is a fatal neurodegenerative disease that accounts for 60–80% of dementia cases worldwide (Boughey and Graff-Radford, 1987; DeTure and Dickson, 2019). The progressive nature of the disease makes for deficits in one or more cognitive domains to occur beyond what one might be expected to incur from normal aging (Boughey and Graff-Radford, 1987). Alzheimer’s disease exists on what is known as the “Alzheimer’s disease spectrum” (ADS), which is characterized by three distinct stages (Yamasaki and Tobimatsu, 2018): (1) The preclinical stage, wherein abnormal lesions peculiar to AD pathology appear across the brain, but clinical symptoms are not yet present (Dubois et al., 2016; Khan, 2018). (2) The mild cognitive impairment (MCI) stage, wherein objective memory impairment takes place without impeding a person’s daily functioning (Neugroschl and Wang, 2011). (3) The AD dementia stage, wherein clinical manifestations of dementia impede a person’s ability to function normally and independently. The clinical detection and diagnosis of AD occur in its end phase, by which time the disease has already taken its course and caused significant brain damage (Schultz et al., 2004).

Pathophysiological AD hallmarks such as extracellular amyloid-beta protein (Aβ) plaques and intracellular aggregation of tau proteins, among others, play a role in the onset of AD (Saint-Aubert et al., 2017; Kim et al., 2021). In particular, tau proteins form into paired helical filaments (PHFs), which ultimately leads to the generation of neurofibrillary tangles (NFTs) that disrupt healthy neuronal function and contribute to the eventual death of the neuron (Palop et al., 2006; Seeley et al., 2009; Wolfe, 2012; Kandel, 2013; Bloom, 2014; Jones et al., 2016; Saint-Aubert et al., 2017; Drzezga, 2018; DeTure and Dickson, 2019). NFTs tend to form and propagate in stereotypical spatio-temporal patterns, which are defined according to the Braak staging scheme (Braak and Braak, 1991; Saint-Aubert et al., 2017; DeTure and Dickson, 2019). Throughout the disease, the repeated and sustained loss of neurons results in structural changes to the brain, wherein we observe shrinkage in the cerebral cortex (gray matter), a process known as cerebral atrophy.

The interplay between the NFTs and the ever-changing anatomical characteristics of the brain serve as important indices of AD-related neurodegeneration. Neuroimaging modalities such as T1-weighted magnetic resonance imaging (MRI) and tau positron emission tomography (PET) play a central role in capturing and quantifying the abovementioned features (Ewers et al., 2011; Márquez and Yassa, 2019). MRI and tau PET produce important measures that quantify disease progression, and they offer complementary information regarding the disease process.

Resting-state functional connectivity studies have led to understanding AD pathology from a different spatial scale with respect to the brain’s functional and structural organization. Functional brain networks are composed of brain regions that exhibit correlated fluctuations in their resting state activity, though they may be physically distributed in space (van den Heuvel and Hulshoff Pol, 2010; Seitzman et al., 2019). Recent hypotheses have proposed an alternate view to the stereotypical anatomical propagation of neuropathological tau in Alzheimer’s disease as suggested by Braak & Braak. Indeed, the network degeneration hypothesis (NDH) suggests that neurodegenerative disorders, such as Alzheimer’s disease, progress along functional networks, ultimately leading to a cascade-like failure of these networks (Drzezga, 2018)–(Seeley et al., 2009). The NDH therefore suggests that tau pathology evolves along functionally connected brain regions. This establishes functional networks as important structures to take into account in a statistical model of the AD neurodegenerative process.

While the Braak staging scheme offers region-wise description of tau propagation, the NDH offers a network-wise description of tau propagation. Though they may be competing views, they nonetheless offer two avenues on how to go about classifying AD stages along the spectrum. The clustering of brain regions into overarching functional networks gives rise to a hierarchical description of the brain’s organization. It becomes evident through the latter description of the brain’s organization that interdependencies exist across elements at different spatial scales. As such, this provides an opportunity to take a consolidative approach to AD classification. That is, to develop statistical models that consider both regions of interest (ROI) and network-level information. However, undertaking such a task necessitates a versatile framework that enables us to construct, evaluate, and carry out our investigations in a principled and robust manner.

The many advances in the Bayesian approach to data analysis have made working with Bayesian models more accessible and efficient. Indeed, the advent of powerful sampling algorithms such as Hamiltonian Monte Carlo (HMC) and the development of a principled methodology known as the Bayesian workflow have greatly facilitated probabilistic analysis under the Bayesian paradigm (Betancourt, 2017; Gelman et al., 2020). To this end, we employed a hierarchical modeling (partial-pooling) strategy within the Bayesian inference framework to investigate whether a statistical classification model’s prediction would benefit from having both ROI-level and network-level information in its model specification. More specifically, we developed four Bayesian hierarchical multinomial logistic regression models, each with increasing levels of complexity in terms of describing the data-generating process. We hypothesized that a model that incorporated ROI-level and network-level would have a better ability to perform classification across diagnostic groups as compared to all other competing models. The hierarchical Bayesian modeling strategy was employed to represent the underlying hierarchical structure of the data and to account for sources of heterogeneity inherent to the observed data. Additionally, a hierarchical structure profits from the effects of partial pooling. Partial pooling enables the sharing of information across clusters and levels to improve parameter estimates through a process known as shrinkage, a regularization mechanism that is a consequence of the hierarchical structure (Gelman, 2014; McElreath, 2020). We assessed each model’s out-of-sample predictive ability and goodness of fit using the leave-one-out cross-validation and posterior predictive check procedures (Gelman et al., 2013; Vehtari et al., 2017).

## 2. Materials and methods

### 2.1. Participants

The data for this study was provided by McGill University’s Research Centre for Studies in Aging (MCSA). The data set is part of the Translational Biomarkers in Aging and Dementia (TRIAD) cohort.^1^ One hundred and thirteen participants were included in the data set, of which 72 (26 males, 46 females) were cognitively normal (CN), twenty-three (11 males, 12 females) had mild cognitive impairments (MCI), and 18 (8 males, 10 females) had AD. Subjects had undergone a baseline, and follow-up structural MRI and tau-PET scan with the median (inter-quartile range) interval between visits was 378 days (356–440) and 418 days (360–476) for MRI and tau-PET, respectively. The participants undertook detailed cognitive and clinical assessments, including the Clinical Dementia Rating (CDR) and Mini-Mental State Examination (MMSE) and neuropsychological tests for memory, attention, executive function, visuospatial processing, psychomotor speed processing and language. Taking into account the clinical presentation, physical examination and aforementioned tests, a multidisciplinary team, including physicians, nurses and neuropsychologists, provided a consensus diagnosis for all participants. Cognitively unimpaired individuals showed no objective cognitive impairment, had a CDR score of 0, and were asked to report any subjective cognitive decline in a questionnaire given during screening. Patients with MCI had subjective and objective cognitive impairment, relatively preserved activities of daily living, and a CDR score of 0.5. Patients with mild-to-moderate sporadic Alzheimer’s disease dementia had a CDR score of between 0.5 and 2 and met the National Institute on Aging and the Alzheimer’s Association criteria for probable Alzheimer’s disease as determined by a physician. Table 1 shows a detailed clinical and demographic description of the cohort.

**Table 1:** Subjects’ demographic information.

	Diagnostic group		
Demographics	AD, *N* = 18^1^	CN, *N* = 72^1^	MCI, *N* = 23^1^
Sex			
F	10 (56%)	46 (64%)	12 (52%)
M	8 (44%)	26 (36%)	11 (48%)
Age	66 (62, 71)	73 (68, 77)	76 (69, 79)
BORB	27.00 (24.50, 29.50)	31.00 (30.00, 31.00)	31.00 (29.00, 31.00)
Boston	24.00 (15.50, 27.00)	30.00 (29.00, 30.00)	28.00 (25.00, 30.00)
D-KEFS	16 (7, 20)	38 (33, 44)	32 (27, 38)
LMDS	0 (0, 0)	17 (15, 19)	9 (6, 13)
LMIS	2 (0, 4)	18 (15, 20)	13 (8, 15)
MMSE	22.0 (13.0, 23.0)	30.0 (29.0, 30.0)	29.0 (27.0, 29.0)
MOCA	13.0 (4.0, 14.0)	28.0 (26.0, 29.0)	25.0 (23.8, 27.0)
WASI-II	82 (76, 88)	113 (106, 122)	106 (94, 111)

^1^n (%); Median (IQR).

BORB, Birmingham Object Recognition Battery; Boston, Boston Naming Test; D-KEFS, Delis-Kaplan Executive Function System; LMDS, Logical Memory Delayed Score; LMIS, Logical Memory Immediate Score; MMSE, Mini Mental State Examination; MoCA, Montreal Cognitive Assessment; WASI-II, Wechsler Abbreviated Scale of Intelligence, Second Edition.

### 2.2. Image acquisition and preprocessing

PET data were acquired using a Siemens High-Resolution Research Tomograph (Therriault et al., 2020). ^18^F-MK-6240 was selected for *in vivo* quantification of tau NFTs due to its high affinity of binding to pathological tau in clinical AD populations (Therriault et al., 2020; Pascoal et al., 2021). Preprocessing was carried out using the protocols described in Therriault et al. (2020) and the inferior cerebellar gray matter was used as a reference region to generate the ^18^F-MK-6240 standardized uptake value ratio (SUVR) images (Therriault et al., 2020). Structural MRI data were acquired on a 3T Siemens Magnetom using a standard head coil (Therriault et al., 2021). Preprocessing was carried out using the protocols described in Therriault et al. (2021) with cortical thickness measures extracted using Freesurfer (v6.0) (Fischl, 2012; Therriault et al., 2021). The Desikan-Killiany-Tourville (DKT) and the Freesurfer subcortical atlases were used to define the regions of interest (Fischl, 2012; Klein and Tourville, 2012). Cortical thickness and SUVR measures were extracted for 62 cortical regions defined by the DKT atlas. Additionally, SUVR measures were extracted for 14 subcortical regions defined by the Freesurfer atlas for a total of 76 ROIs. The DKT cortical ROIs were also mapped to the Yeo 7 resting-state networks (Thomas Yeo et al., 2011; Bhagwat et al., 2021). The functional networks Yeo 7 explored the organization of large-scale distributed networks in the human cerebral cortex using resting-state functional connectivity MRI. A clustering approach was employed to identify and replicate networks of functionally coupled regions across the cerebral cortex. There are several bordering regions belonging to different large-scale networks. Functional network parcellations of the cerebral cortex into 7 networks (i.e., control, default, dorsal attention, somatomotor, visual, ventral attention and limbic networks) were considered in this paper. Subcortical ROIs are assumed to be clustered at the network level as an additional entity since a well-established mapping of said ROIs are not defined. The ROIs feature the Desikan-Killiany-Tourville cortical and Freesurfer subcortical ROI labels (1–38), each matched with their corresponding Yeo 7 resting-state networks (1–7) for the right hemisphere. The assignment of ROIs to Yeo 7 network is provided by the parcellation of the Yeo 7 network. The details are shown in Table 2.

**Table 2:** Desikan-Killiany-Tourville cortical and Freesurfer subcortical ROI labels (1–38) present in the data set as well as their correspondence to the Yeo 7 resting-state networks (1–7) for the right hemisphere.

	Yeo 7 Network	Braak stage
Subcortical (SUBC)		
[1] Hippocampus (HIPP)	–	Stage 2
[3] Amygdala (AMYG)	–	Stage 3
[4] Thalamus Proper (THALP)	–	
[5] Caudate (CAUD)	–	
[6] Putamen (PUT)	–	
[7] Pallidum (PAL)	–	
[8] Accumbens Area (ACUM)	–	
Temporal Lobe (TEMP)		
[2] Entorhinal (ENT)	[5] Limbic (LIM)	Stage 1
[12] Fusiform (FUS)	[1] Visual (VIS)	Stage 3
[14] Inferior Temporal (IT)	[5] Limbic (LIM)	Stage 4
[20] Middle Temporal (MT)	[7] Default Mode (DM)	Stage 4
[21] Parahippocampal (PARH)	[7] Default Mode (DM)	Stage 3
[35] Superior Temporal (ST)	[2] Somatomotor (SOM)	Stage 5
[37] Transverse Temporal (TT)	[2] Somatomotor (SOM)	Stage 3
Cingulate Cortex (CING)		
[9] Caudal Anterior Cingulate (CAC)	[4] Salience/Ventral Attention (SAL)	Stage 4
[15] Isthmus Cingulate (ISTC)	[7] Default Mode (DM)	Stage 4
[28] Posterior Cingulate (PC)	[7] Default Mode (DM)	Stage 4
[31] Rostral Anterior Cingulate (RAC)	[7] Default Mode (DM)	Stage 4
[38] Insula (INS)	[4] Salience/Ventral Attention (SAL)	Stage 4
Frontal Lobe (FRNT)		
[10] Caudal Middle Frontal (CMF)	[6] Control (CTL)	Stage 5
[17] Lateral Orbitofrontal (LORB)	[5] Limbic (LIM)	Stage 5
[19] Medial Orbitofrontal (MORB)	[5] Limbic (LIM)	Stage 5
[22] Paracentral (PARC)	[2] Somatomotor (SOM)	Stage 6
[23] Pars Opercularis (POPE)	[4] Salience/Ventral Attention (SAL)	Stage 5
[24] Pars Orbitalis (PORB)	[7] Default Mode (DM)	Stage 5
[25] Pars Triangularis (PTRI)	[7] Default Mode (DM)	Stage 5
[29] Precentral (PREC)	[2] Somatomotor (SOM)	Stage 6
[32] Rostral Middle Frontal (RMF)	[6] Control (CTL)	Stage 5
[33] Superior Frontal (SF)	[7] Default Mode (DM)	Stage 5
Occipital Lobe (OCC)		
[11] Cuneus (CUN)	[1] Visual (VIS)	Stage 6
[16] Lateral Occipital (LOCC)	[1] Visual (VIS)	Stage 5
[18] Lingual (LIN)	[1] Visual (VIS)	Stage 3
[26] Pericalcarine (PCAL)	[1] Visual (VIS)	Stage 6
Parietal Lobe (PAR)		
[13] Inferior Parietal (INFP)	[7] Default Mode (DM)	Stage 5
[27] Postcentral (PSTS)	[2] Somatomotor (SOM)	Stage 6
[30] Precuneus (PCUN)	[7] Default Mode (DM)	Stage 5
[34] Superior Parietal (SP)	[3] Dorsal Attention (DA)	Stage 5
[36] Supramarginal (SMAR)	[4] Salience/Ventral Attention (SAL)	Stage 5

The abbreviation for each label is also shown. Although omitted from the table, left hemisphere mappings are identical, with the cortical and subcortical ROI labels ranging from (39–76). Moreover, ROI correspondence to a given Braak stage is also shown.

### 2.3. Metrics to assess disease progression

For every subject, the annualized rate of change in ^18^F-MK-6240 SUVR in each ROI was computed as the difference between the follow-up and baseline SUVR uptake values within a given ROI divided by the time interval between scans in years (Equation 1):


(1) $$\left(\frac{{\mathrm{SUVR}}_{Follow-up}-{\mathrm{SUVR}}_{Baseline}}{\Delta \mathrm{time}}\right)$$

The relative annualized rate of change in ^18^F-MK-6240 SUVR was computed by dividing the annualized rate by the baseline SUVR uptake (Equation 2):


(2) $$\left(\frac{{\mathrm{SUVR}}_{Follow-up}-{\mathrm{SUVR}}_{Baseline}}{\Delta \mathrm{time}}\right)\times \left(\frac{1}{{\mathrm{SUVR}}_{Baseline}}\right)$$

Similarly, relative annualized rate of change in cortical thickness for each ROI was computed as (Equation 3):


(3) $$\begin{array}{l} -\left(\frac{\mathrm{Cortical}\;{\mathrm{Thickness}}_{Follow-up}-\mathrm{Cortical}\;{\mathrm{Thickness}}_{Baseline}}{\Delta \mathrm{time}}\right)\\ \times \left(\frac{1}{\mathrm{Cortical}\;{\mathrm{Thickness}}_{Baseline}}\right) \end{array}$$

The minus sign in front equation 3 was added to help interpret the metric more intuitively. A positive rate indicates more cortical thinning, whereas a negative rate indicates cortical growth.

### 2.4. Bayesian hierarchical models

The Bayesian inference and modeling paradigm are used to fit a probability model to a data set and explicitly quantify uncertainty about parameters of interest through probability distributions (Gelman et al., 2013). Given that the task was classification for multiple classes, it was fitting to implement a multinomial regression model. Let us begin by providing some definitions to the multinomial likelihood function (Equation 4):


$$y_{i}=\mathrm{Multinomial}\left(\pi_{ij}\right)\;\;\left[\mathrm{likelihood}\right]$$

$$\pi_{ij}=p\left(y_{i}=j|\lambda^{j}\right)$$

where


$$\lambda^{\left(j\right)}=\beta_{0}^{\left(j\right)}+\overset{K}{\underset{k=1}{\sum }}\beta_{k}^{\left(j\right)}x_{k}\;\;\left[\mathrm{linear model}\right]\;\;\;\;\;\;\;\;\;\;\;\;(4)$$

where $y_{i}$ is a response variable for the ith observation that follows a multinomial likelihood and can take on a given nominal value from the set of categorical outcomes j∈{1,2,…,J} . We let $\pi_{ij}$ denote the probability that the ith observation will fall into the jth category conditional on the underlying linear propensity of the jth outcome. $\beta_{0}$ is the intercept parameter and k denotes the index of the K predictor variables $x_{k}$ and their corresponding slope parameters $\beta_{k}$ in the linear model. Superscripted terms in between round brackets indicate their association to the jth categorical outcome and to reduce confusion with other subscripts in the linear model. Furthermore, the softmax function, a generalization of the logistic function, is used as the inverse link function to map the linear model to the outcome scale (Equation 5):


$$\pi_{ij}={\mathrm{softmax}}_{J}\left(\left\{\lambda^{\left(j\right)}\right\}\right)$$

where


$${\mathrm{softmax}}_{J}\left(\left\{\lambda^{\left(j\right)}\right\}\right)=\frac{\exp\left(\lambda^{\left(j\right)}\right)}{\sum_{m=1}^{J}\exp\left(\lambda^{\left(m\right)}\right)}\;\;\;\;\;(5)$$

The equation states that the probability of the ith observation falling in the jth category is the obtained by exponentiating the linear propensity of the jth outcome and normalizing it by the sum of exponentiated linear propensities across the set of all possible categorical outcomes (Barry, 2011).

**Figure 1:**
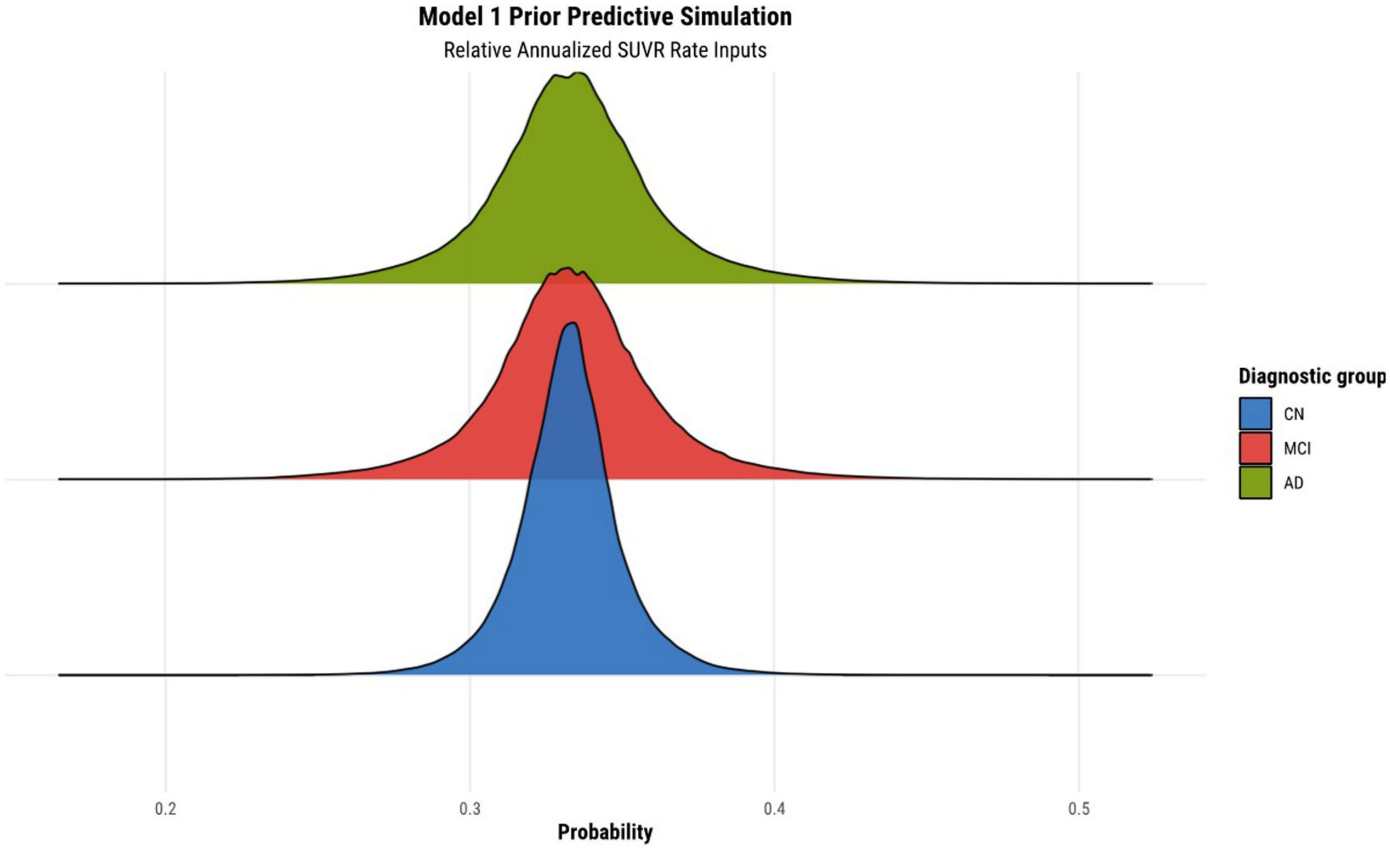
Prior predictive simulation for model 1 using relative annualized SUVR as input. The *x*-axis of the plot indicates the probability of being classified in a particular diagnostic group. The distributions are color coded according to the corresponding diagnostic group, where the green, red, and blue colors represent the AD, MCI, and CN diagnostic groups, respectively.

Before fitting the models, we normalized the relative annualized SUVR and cortical thickness rates to keep the range of inputs between −1 and 1. The latter normalization further enabled us to set priors more easily within the models. The brms package (Version 2.17.0) in R (Version 4.2.1) was used to specify the models, prior predictive simulations, and generate samples from their posterior distributions *via* Hamiltonian Monte Carlo (HMC), an efficient Markov chain Monte Carlo (MCMC) sampling method (Bürkner, 2017). Results were based on running four parallel chains with 2000 total iterations per chain, including 1,000 warmup (burn-in) iterations, for a total of 4,000 post-warmup draws. To validate our choice of priors for the parameters of each model, we conducted a prior predictive simulation. We manually tuned the values of the priors to ensure an objective output distribution. The result of the prior predictive simulation for model 1 can been seen in Figure 1, illustrating the desirable qualities that were also exhibited by all other subsequent model simulations once their respective priors were tuned.

#### 2.4.1. Hierarchical Bayesian model #1: complete pooling model

Model 1 is the most basic model wherein we assume that all data points stem from a single overarching data distribution. The mathematical description of model 1 is as follows (Equation 6):


(6) $$\begin{array}{c} y_{i}∼\mathrm{Multinomial}\left(\pi_{ij}\right)\;\;\left[\mathrm{likelihood}\right]\\ \pi_{ij}=\frac{\exp\left(\lambda^{\left(j\right)}\right)}{\sum_{m=1}^{3}\exp\left(\lambda^{\left(m\right)}\right)}\;\;\left[\mathrm{inverse}-\mathrm{link function}\right]\\ \begin{array}{c} \lambda^{\left(1\right)}=0\;\left[\mathrm{reference linear model}\;\mathrm{CN}\right]\\ \lambda^{\left(2\right)}=\beta_{0}^{\left(2\right)}+\beta_{1}^{\left(2\right)}x_{1}\;\;\left[\mathrm{linear model}\;\mathrm{MCI}\right]\\ \begin{array}{c} \lambda^{\left(3\right)}=\beta_{0}^{\left(3\right)}+\beta_{1}^{\left(3\right)}x_{1}\;\;\left[\mathrm{linear model}\;\mathrm{AD}\right]\\ \begin{array}{c} \beta_{0}^{\left(j\right)}∼\mathrm{Normal}\left(0,0.05\right)\;\;\left[\mathrm{intercept prior}\right]\\ \beta_{1}^{\left(j\right)}∼\mathrm{Normal}\left(0,0.2\right)\;\;\left[\mathrm{slope prior}\right] \end{array} \end{array} \end{array} \end{array}\;$$

The input to the model is either the tau-PET rates or cortical thickness rates. The multinomial model requires a categorical reference outcome, and as such, the CN category was designated as the reference category. All parameters in the model require the specification of a prior distribution. The Gaussian prior was assigned to both the intercept and slope parameters since it properly reflects our state of knowledge regarding the regression parameters: they will lie within a plausible range of values consistent with our understanding of the pathophysiological process of the disease. In other words, assuming the parameters have finite variance warrants the use of the Gaussian distribution, as it represents the most objective and conservative probability distribution consistent with our partial scientific knowledge of our parameters (McElreath, 2020).

#### 2.4.2. Hierarchical Bayesian model #2: observations within ROIs

Model 2 implements a hierarchical structure at a basic level. In this model, subjects’ observations are clustered within ROIs. Each ROI’s distributional parameters are assumed to come from an overarching probability distribution. The mathematical description of model 2 is as follows (Equation 7):
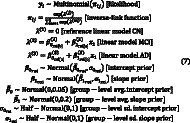


In model 2, both the intercept and slope parameters within the linear MCI and AD linear models are indexed by a subscript u in square brackets. This notation emphasizes that the inputs (relative annualized SUVR or cortical thickness) to the model are labeled according to their membership of a particular ROI. In the case of SUVR data, u ranges from 1 to 76. In the case of cortical thickness data, u ranges from 1 to 62. Next, the priors at the ROI level are described by Gaussian distributions specified by parameters as input arguments. This follows from the assumption that observations are mutually representative of the clusters they are grouped in (in this case, a given ROI). The group-level parameters are also estimated and given a set of priors. Since the group-level parameters are described by a Gaussian distribution, there needs to be a specification of the prior for both the mean and the standard deviation. These group-level priors are specified in the four last lines of Equation 7. The group-level prior for the standard deviation is chosen to be a Half-Normal distribution since variance is constrained to be positive. Although choices such as the exponential distribution are also common as a choice of prior, the Half-Normal was chosen due to its gradual decay rate compared to the exponential distribution.

#### 2.4.3. Hierarchical Bayesian model #3: observations within functional networks

In this model, subjects’ observations are clustered within networks. Each network’s distributional parameters are assumed to come from an overarching probability distribution. Similar to model 2’s subscript notation, the subscript v indicates membership of a particular network. The mathematical description of model 3 is as follows (Equation 8):
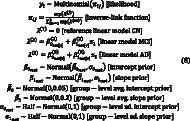


In the case of SUVR data, v ranges from 1 to 8. As was previously mentioned, subcortical ROIs are assumed to be clustered at the network level as an additional entity. In the case of cortical thickness data, u ranges from 1 to 7.

#### 2.4.4. Hierarchical Bayesian model #4: observations within ROIs, within functional networks

Model 4 implements the complete hierarchical description of the data-generating process. In this model, subjects’ observations are clustered within ROIs that are also clustered within functional networks. As such, the distributional parameters of each ROI are assumed to come from a specific overarching network distribution, which in turn is assumed to come from an overarching distribution for all networks. The mathematical description of model 4 is described below in an alternate but equivalent form for simplicity (Equation 9):
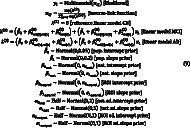


The level 4 model can be seen as the level 3 model, with an additional ROI-level intercept parameter $\beta_{0_{\mathrm{net}\left[v\right],\mathrm{roi}\left[u\right]}}^{\left(j\right)}$ and ROI-level slope parameter $\beta_{1_{\mathrm{net}\left[v\right],\mathrm{roi}\left[u\right]}}^{\left(j\right)}$ . The indexing subscripts indicate that ROIs are specific to a given network. As such, these parameters describe ROI-specific deviations within a given network and are assigned a set of priors.

### 2.5. Model evaluation and comparison

#### 2.5.1. Posterior predictive checks

A method used to assess model adequacy and the fit of the model to the data is the posterior predictive check (PPC) (Vehtari et al., 2017). The idea behind the PPC is the following: if a model adequately represents the underlying data generating process, then it should be able to generate data that resemble the initial data observations (Gelman et al., 2013). To conduct a PPC, we first select a model fit to the data. We then draw many replications (e.g., 1,000) from the parameters in the fitted model to create simulated data sets whose size is equal to that of the observed data set. We then visually compare the observed data set and the replicated data sets and make an assessment of any discrepancies. Any major discrepancies between the simulated data and the observed data indicate potential model misspecification (Gelman et al., 2013). The posterior predictive checks for all four models were performed for both relative annualized SUVR and cortical thickness rates inputs.

#### 2.5.2. Leave-one-out cross-validation

Another evaluation criteria to assess model adequacy as well as predictive performance is leave-one-out cross-validation. In essence, it is a method used to determine how well a model generalizes to new data. Cross-validation is a well-known strategy to estimate a given model’s out-of-sample predictive accuracy. At times, we may not have new data to use as an input to a model. However, it is possible to partition the initial data set into a set with which we train and fit the model and use the remaining data to evaluate model performance. Bayesian evaluation techniques utilize leave-one-out cross-validation (LOO-CV), wherein the training set contains all but one of the samples and the remaining point is used as a test set (Gelman et al., 2013). All four models’ cross validation scores were assessed for both relative annualized SUVR and cortical thickness rates inputs. Models were then ranked from worst to best in terms of the Bayesian estimate of the leave-one-out expected log point-wise predictive density ( ${\mathrm{elpd}}_{\mathrm{loo}}$ ) value.

### 2.6. Posterior predictions

Given a fitted model, posterior predictions can be made given a set of input data. Though posterior predictions could be performed using each model, we chose to perform the predictions using the winning model determined from the results of the evaluation and comparison steps. We simulated predictions for relative annualized SUVR and cortical thickness rate inputs. We selected the inputs to range between the observed minimum and maximum relative annualized rates of SUVR and cortical thickness across the entire data set, respectively. The range is between −0.42 and 0.71 for tau-PET, and between −0.26 and 0.23 for cortical thickness.

### 2.7. Data and code availability statements

The data used in this study (see text footnote 1) was provided by the McGill University’s Research Centre for Studies in Aging (MCSA) and is available upon reasonable request to the corresponding authors. The corresponding R code for brms models is available upon reasonable request to the corresponding author.

## 3. Results

### 3.1. Prior predictive simulation

The results of the prior predictive simulation of model 1 using relative annualized SUVR as an input is shown in Figure 1. The distributions of the prior predictive simulation for model 1 shows that all three diagnostic groups are centered around 0.33 with minimal spread. This means that the prior implies fairness being classified into any of the three diagnostic outcomes. In other words, in the absence of any evidence (the data observations), the priors of the model treat all outcomes as equally likely. As such, our model does not show any bias or preference to any one diagnostic group *a priori*. As was mentioned previously, similar simulations were conducted for all other hierarchical models to ensure that the models did not have any *a priori* bias of classification toward a particular diagnostic group.

### 3.2. Model diagnostics

All models were successfully fit with no reported divergent transitions from the brms diagnostics. The results indicate the models were properly parametrized and that the HMC chains had successfully explored the target distribution, which is further validated by an $\hat{R}$ < 1.05 asserting the chains’ convergence (Betancourt, 2017; Gelman et al., 2020). Moreover, the diagnostics for Pareto smoothed importance sampling (PSIS) for leave-one-out cross-validation showed that all Pareto shape parameter k estimates are good (*k* < 0.5), indicating that the ${\mathrm{elpd}}_{\mathrm{loo}}$ was estimated with high accuracy. Moreover, the Monte-Carlo standard error (MCSE) assessing the computational accuracy of the Markov-Chain Monte-Carlo and importance sampling used to compute the ${\mathrm{elpd}}_{\mathrm{loo}}$ was between 0.0 and 0.1 across all models (Vehtari et al., 2015; Gabry et al., 2019).

### 3.3. Posterior predictive checks

**Figure 2:**
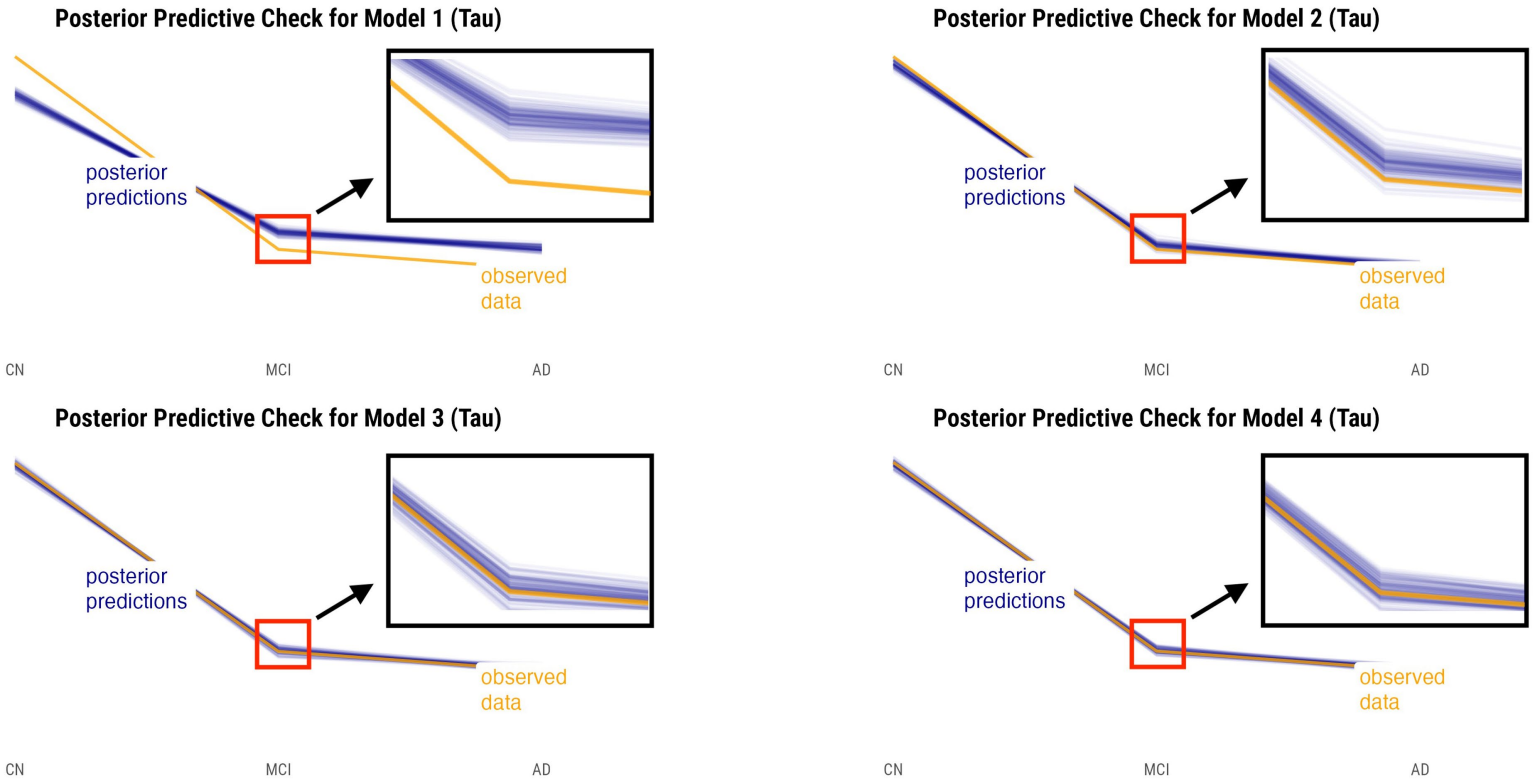
Posterior predictive checks for models 1–4 with relative annualized SUVR as the predictor. The orange line represents the observed data for each diagnostic group, whereas the blue lines represent the posterior predictive replications.

**Figure 3:**
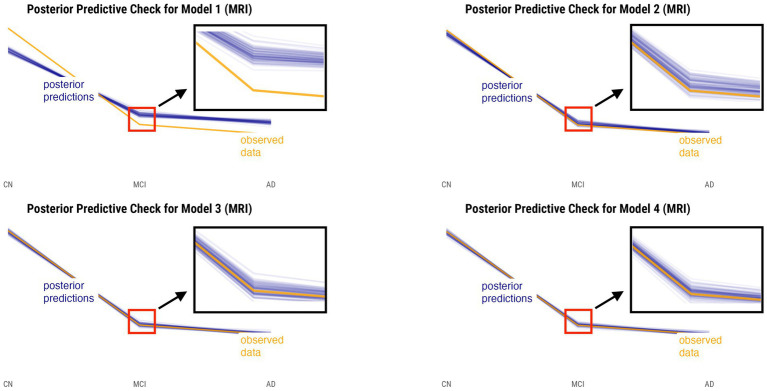
Posterior predictive checks for models 1–4 with relative annualized cortical thickness as the predictor. The orange line represents the observed data for each diagnostic group, whereas the blue lines represent the posterior predictive replications.

Posterior predictive checks were performed for all models. The PPCs for models 3 and 4 seem to have a better ability to replicate the initial data better than models 1 and 2. Between the models 3 and 4, model 4 seems to be able to best generate data that resemble the initial data observations. This is shown by model 4’s simulated replications having the tightest spread around the center of the observed data for both relative annualized SUVR and cortical thickness rate inputs, as compared to the other models’ PPC plots. The posterior predictive checks for all four models for both tau-PET and cortical thickness predictors (referred to as tau and MRI for brevity) are shown in Figures 2, 3, respectively. For both figures, the orange lines depict the observed data while the blue lines indicate the posterior predictive replications.

### 3.4. Leave-one-out cross-validation

The leave-one-out cross-validation results indicate that models 3 and 4, models that both incorporate network level information, outperform models 1 and 2 for tau-PET and cortical thickness inputs. Model 3 is the best model for tau-PET inputs while model 4 is the best model for cortical thickness inputs. The results for all models given either the relative annualized SUVR or cortical thickness rates are displayed in Table 3. The table displays the out-of-sample performance for each model relative to model with the lowest ${\mathrm{elpd}}_{\mathrm{loo}}$ value (third column) that is placed at the first row for each of the inputs. As such, the difference in elpd (first column) and difference in standard error (second column) are displayed relative to the model with the lowest ${\mathrm{elpd}}_{\mathrm{loo}}$ value for each input. The fourth column shows the standard error of the ${\mathrm{elpd}}_{\mathrm{loo}}$ value for each model.

**Table 3:** Leave-one-out cross-validation results for all four statistical models given either the relative annualized SUVR (top half of table) or cortical thickness (bottom half of table) rates.

	elpd_diff	se_diff	elpd_loo	se_elpd_loo
Tau-PET Models				
Model 3 (Network only)	0.000000	0.000000	−7542.332	60.51120
Model 4 (Network & ROI)	−3.834226	1.303660	−7546.166	60.53048
Model 2 (ROI only)	−151.583124	10.910374	−7693.915	58.34978
Model 1 (Complete-pooling)	−209.893005	23.797981	−7752.225	40.27197
Cortical Thickness Models				
Model 4 (Network & ROI)	0.000000	0.000000	−6247.263	51.25579
Model 3 (Network only)	−29.832498	7.554926	−6277.096	51.02425
Model 2 (ROI only)	−79.467189	4.115720	−6326.731	49.94705
Model 1 (Complete-pooling)	−188.390642	19.481006	−6435.654	33.98546

The first two columns express the difference in elpd as well as standard error relative to the model will the lowest ${\mathrm{elpd}}_{\mathrm{loo}}$ value (third column). The fourth column shows the standard error of the ${\mathrm{elpd}}_{\mathrm{loo}}$ value for each model.

### 3.5. Posterior predictions

**Figure 4:**
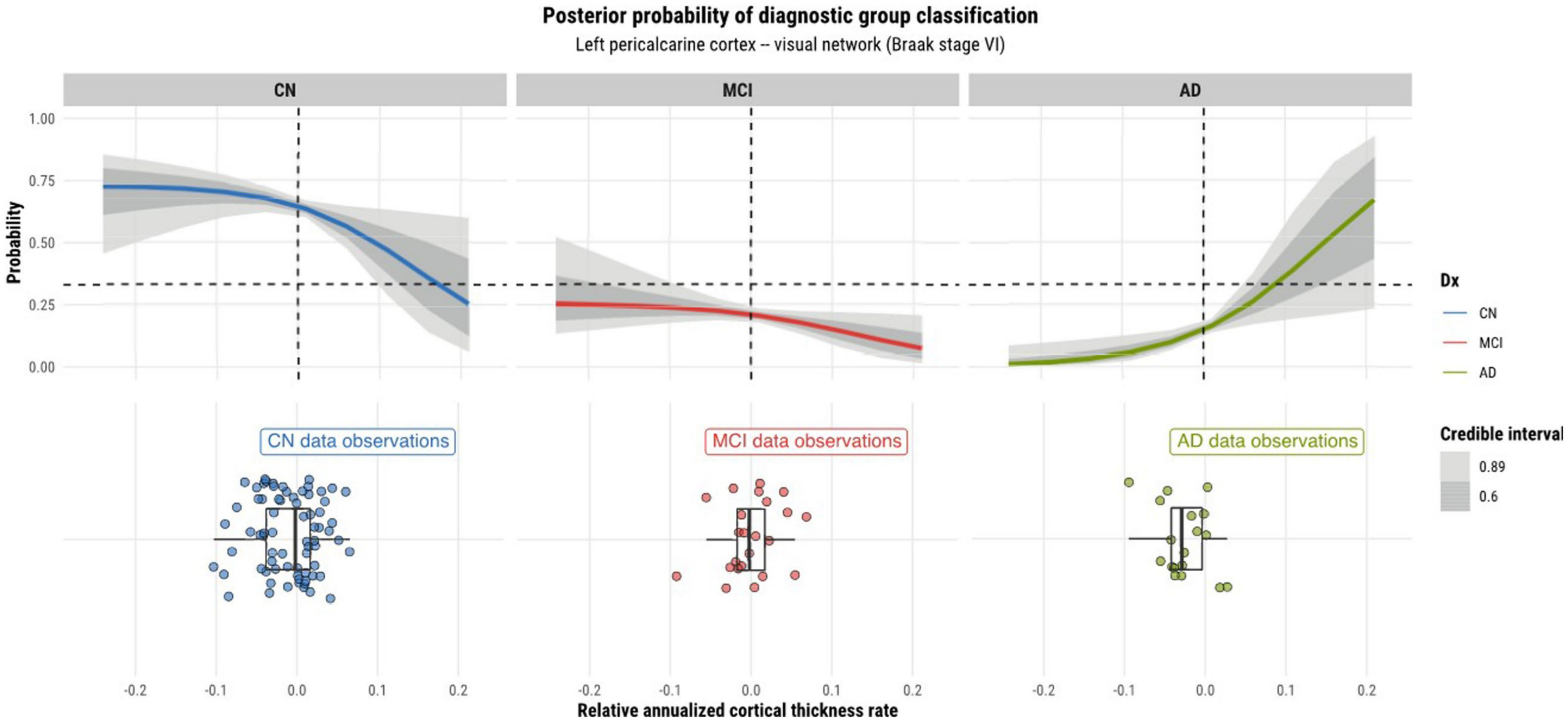
Model 4 posterior predictions with prediction uncertainty for the pericalcarine cortex in the left hemisphere of the brain given relative annualized cortical thickness rates as an input.

**Figure 5:**
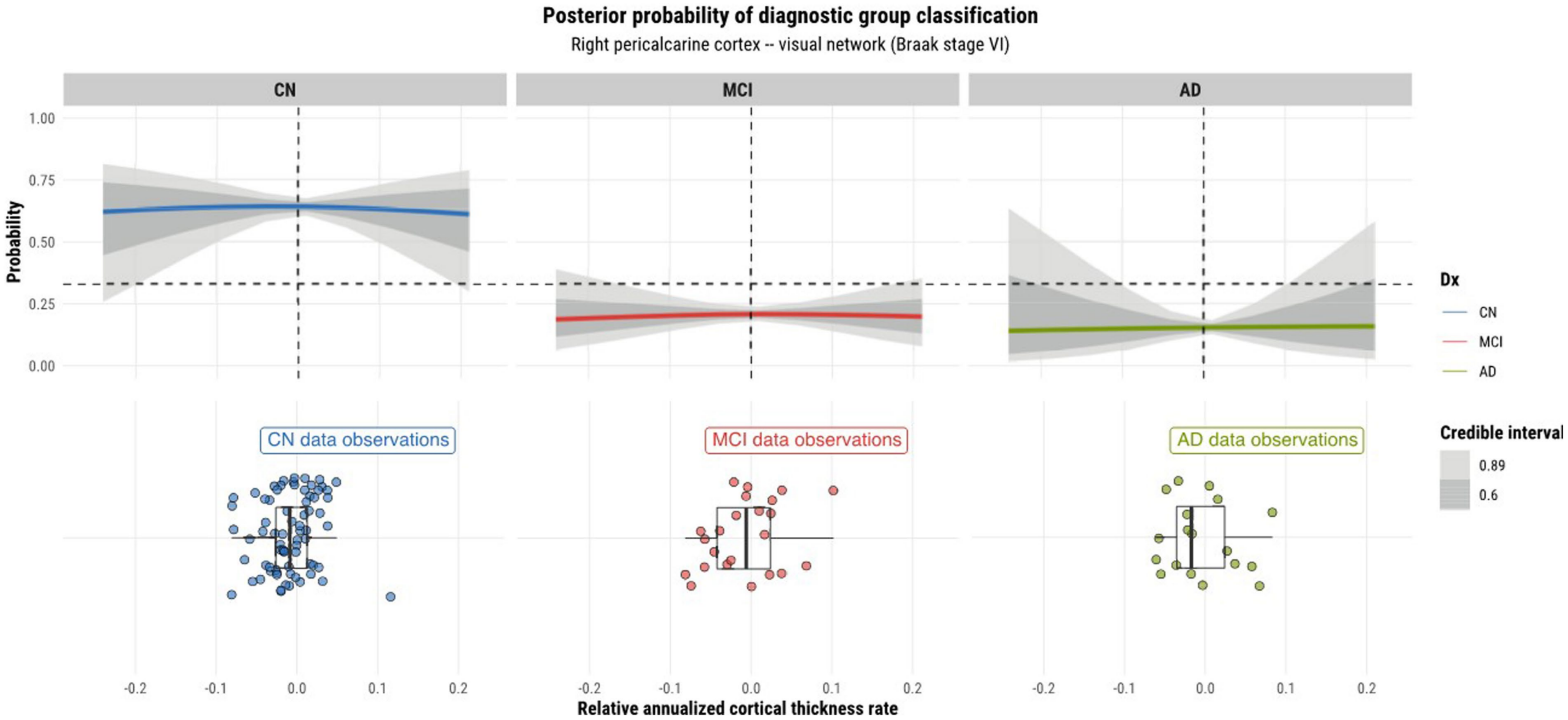
Model 4 posterior predictions with prediction uncertainty for the pericalcarine cortex in the right hemisphere of the brain given relative annualized cortical thickness rates as an input.

Model 4 posterior probabilities for the pericalcarine cortex within the left and right hemispheres are displayed in Figures 4, 5, respectively, revealing laterality in prediction between hemispheres. The top panel of the plots shows the posterior probabilities of the region being classified into a particular diagnostic group for a range of relative annualized cortical thickness rates. The solid curve represents the mean posterior probability with the uncertainty in prediction shown by 60 and 89% credible intervals around the mean prediction. The bottom panel shows the distribution of the data observations for each diagnostic group in the form of a box plot overlaid with a jitter plot. Moreover, Figure 5 illustrates the posterior probabilities for regions in the visual network (also labeled according to their corresponding Braak stage) within the right brain hemisphere for a range of relative annualized SUVR rates. Mean posterior probabilities as well as the uncertainty around the mean are also shown for each diagnostic group prediction curve. In both figures, the horizontal dashed line shows the points along the *y*-axis where the probability corresponds to 33%. The vertical dashed line shows the point along the *x*-axis where the input is 0.

## 4. Discussion

### 4.1. Model evaluation, comparison, and selection

Using the Bayesian hierarchical modeling strategy and the Bayesian workflow, we aimed to assess whether a model that included both network-level and ROI-level information would improve diagnostic classification performance. Following the Bayesian workflow strategy, we took an iterative model building approach to develop four statistical models with increasing complexity to be able to modularly encapsulate the underlying data-generating process as well. Moreover, by taking an iterative approach to model building, we are able to understand the implications of each model at each stage (Gelman et al., 2020). For instance, given that model 1 is a completely pooled model, it assumes that all data points stem from a single underlying data distribution thereby suppressing any sources of variation specific to particular clusters at various levels within the data hierarchy. As such, a single slope and intercept parameter is estimated and applied globally to all data points. As a result, the model is underfit and any residual error is attributed to measurement noise rather than known sources of variation within the data (Gelman and Hill, 2006). Though models 2 and 3 explicitly model sources of heterogeneity in the data through the specification of a hierarchical structure, they nonetheless lack the ability to perform predictions at both the ROI and network levels simultaneously, as is the case with model 4. Given that model 4 describes the underlying data generating process in a more detailed manner, it benefits from a richer mutual sharing of information across all levels of the model.

We performed posterior checks as well as utilized out-of-sample predictive performance methods to help compare and evaluate the models and ultimately pick the winning model for each input. Referring to Figures 2, 3, we can notice how the PPC plots for models 3 and 4 seem to have an overall better fit to the data for both tau-PET and cortical thickness inputs, as compared to the PPC plots of models 1 and 2. The PPC plot of model 1 displays the highest amount of discrepancy, which is expected given that it is a very basic model that does not take into account the many interdependencies that the partially-pooled models do. The PPC plots for model 3 and 4 show that the simulated replications are increasingly tightly spread around the observed data. Though this visual assessment can serve as a sanity check with regard to the modeling decisions made at every model iteration, we nevertheless need to use an evaluation method that can help us objectively determine a winning model.

Leave-one-out cross-validation results were used to select the best model for each input. Given that it is hard to compare models based on their nominal elpd and standard error values, we take the difference between the two models and look at the difference of their elpd (elpd diff) as well as the standard error (se diff). When comparing multiple models to one another, we compare every other model to the best fitting model according to the cross-validation results. By computing the difference in standard error between two models, we implicitly assume that the sampling distribution of the difference of elpd is asymptotically normal. As a rule of thumb, a difference in standard error less than four indicates that two models have comparable predictive ability (Sivula et al., 2020).

Referring to Table 3, we see that model 4 is deemed the best model for cortical thickness inputs. This assurance comes with the fact that all other models have a value of standard error difference greater than four and large elpd differences from model 4. As for tau-PET inputs, the cross-validation results indicate that model 3 is the best. However, we can notice that model 4 marginally trails behind model 3 by a very small difference in elpd and standard error with the difference in standard error not bring greater than four. Though the ranking of models was based on the nominal elpd values, we still opt for model 4 over model 3 for tau. The reason is that model 4 allows us to perform predictions at both the ROI and network levels, whereas model 3 can only perform network-level predictions. Furthermore, model 4 describes the underlying data generating process in its most complete form, where observations are clustered into ROIs that are subsequently clustered into networks. We regard model 4 to be the best model for both tau-PET and cortical thickness inputs, and confirm that models constructed with both network-level and ROI-level information improve prediction performance.

### 4.2. Model posterior predictions

#### 4.2.1. Laterality of predictions

As model 4 was chosen as the best model for both tau and cortical thickness predictors, we proceeded to using it to perform posterior predictions. Numerous peculiarities were observed from the predictions. Referring to Figures 2, 3, we see that the prediction curves of the left and right hemispheres for the pericalcarine cortex are not in agreement with one another. In particular, we notice a decreasing trend in the CN curve for the left hemisphere as the rate of cortical thinning increases. In contrast, there is an increasing trend in the AD curve as the rate of cortical thinning increases. On the other hand, the right hemisphere does not exhibit the same behavior. The right hemisphere shows a flat-like trend for both CN and AD prediction curves across the range of inputs. We refer to this phenomenon as a leftward-biased lateralization in prediction. Hemispheric asymmetry and lateralization of structure and function in the brain are commonplace in humans and asymmetries can certainly be present in the course of diseases such as AD (Minkova et al., 2017). Moreover, some studies have shown the left hemisphere to be particularly vulnerable in AD and have also reported cortical thickness reductions in areas such as the pericalcarine cortex (Thompson et al., 2001; Yang et al., 2019; Lubben et al., 2021). We also observed a pattern of leftward-biased laterality across the salience network. The salience and default mode networks are two networks that are known to sustain damage from AD (He et al., 2014). Though laterality was not explicitly modeled, it is interesting to observe such patterns emerge in the posterior predictions at both the ROI and network levels.

#### 4.2.2. ROI prediction heterogeneity within functional networks

**Figure 6:**
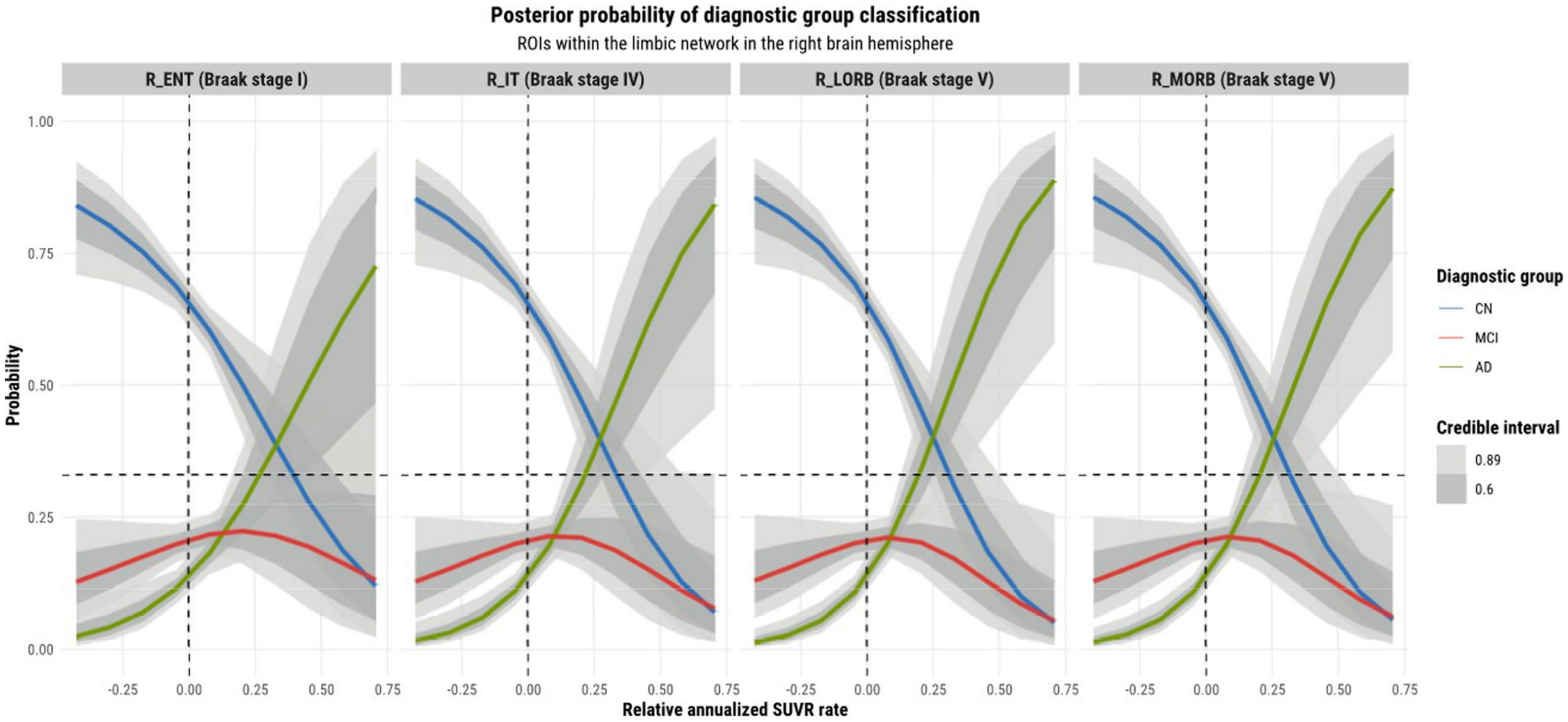
Model 4 posterior predictions with prediction uncertainty for ROIs within the limbic network within the right brain hemisphere with relative annualized SUVR rates as an input. The plots are ordered by increasing Braak stages.

Observations were made on the tau-PET predictions across ROIs within the left and right hemisphere and their relationship with the Braak staging scheme. Referring to Figure 6, we observe a sub-pattern as we go from the entorhinal cortex (stage 1 region) to the medial and lateral orbitofrontal cortices (stage 5 regions). In both hemispheres (but more so in the left hemisphere), we notice an increase in the mean probability of AD, and an increase in specificity for distinguishing it from other diagnostic classes for higher rates of tau SUVR increases as we move further away (in terms of Braak stages) from the entorhinal cortex. The reason for this growing departure in mean AD prediction relative to the entorhinal cortex can be explained by the fact that all other regions within the limbic network are late-stage Braak regions. The entorhinal cortex being a region affected by AD at an early stage may not incur as much change in tau-SUVR from 1 year as it will likely have a reached a plateau earlier on. Tau-SUVR accumulation in late-stage regions within the limbic system have likely not yet plateaued and will have more room to experience a larger swing in tau-SUVR from 1 year to the next. Given that a larger relative annualized rate of change would be expected for late-stage regions, we suspect that this feature is for this reason a clearer discriminator between AD and non-AD classes at the higher range of inputs. More notably, though tau propagation may certainly occur along certain functional networks, the prediction curves display region-specific progression patterns. Although ROIs may be clustered into functional networks, the network as a whole does not progress homogeneously. In other words, ROIs within a particular functional network are not all affected simultaneously.

### 4.3. Limitations and perspectives

One of the limitations of this work was the imbalance in the size of data across diagnostic groups. Though Bayesian methods are well suited to handle small data sets, it would nevertheless be beneficial to have a larger number of subjects in both the MCI and AD groups. Within the Bayesian framework, this would inevitably lead to better inferences for the parameters of the statistical model and better quantification of the uncertainty in model predictions due to a more representative data set.

Another extension of this study could be to develop a model with a different likelihood function to perform classification. Though we used a multinomial logistic regression for our work, we could also treat the underlying diagnostic groups as ordered instead of unordered. The underlying ordering of diagnostic groups would give rise to an ordinal or ordered probit regression, where we would employ a thresholded cumulative normal distribution as the inverse-link function (Barry, 2011).

While we only assessed the advantages of incorporating ROI and network hierarchical structure into classification using a multinomial logistic regression, it is plausible that this hierarchical regularization could prove beneficial for other classification models in the context of AD research, especially considering the variability among the cohort. Our primary objective is to compare model performance across different hierarchical structures. As a result, we opted not to delve into potential clinical applications, for instance, exploring associations between specific network patterns and AD characteristics. Such interpretations of the model require further investigation.

Aging effects may potentially influence the prediction accuracy. In this study, we simply assumed a linear aging effect which can be addressed when computing the annual rate changes of the measure of interest. If focusing on the main message of this study, which highlights the benefits of the hierarchical structure in the prediction model, we believe that these advantages likely persist as long as the measurements across brain regions are not entirely being homogeneous or heterogeneous. Nonetheless, upcoming research should explore the value of this hierarchical structure when incorporating a specific aging effect modeled in the prediction.

## 5. Conclusion

In this work, we aimed to investigate whether a statistical classification model for diagnosing AD could benefit from integrating information at the ROI level and network level in its model specification. The underlying hypothesis was motivated by both the Braak staging scheme and the network degeneration hypothesis. The former describes an ROI-based neurodegeneration pattern while the latter suggests a network-based neurodegeneration pattern. We developed four statistical models using a hierarchical approach within the Bayesian framework to best incorporate the various levels of heterogeneity present in the data. We applied a modular modeling technique, where models were built with increasing levels of complexity. Leave-one-out cross-validation was used to assess out-of-sample model prediction, whereas posterior predictive checks were used to assess the model’s goodness of fit to the observed data. It was then determined that the model that incorporated both ROI-level and network-level information was the best, thus allowing us to confirm our initial investigation hypotheses. In addition to the latter, we observed patterns of physiological interest while performing model predictions. The first pattern was that of laterality, mainly exhibited for cortical thickness inputs. We observed how regions within a particular network differed in their predictions in the case of tau-PET inputs. This suggested that though the disease may globally affect functional networks, the regions that comprise a given network display their own patterns of heterogeneity and progression.

## Data availability statement

Publicly available datasets were analyzed in this study. This data can be found here: The Translational Biomarkers in Aging and Dementia (TRIAD) cohort (https://triad.tnl-mcgill.com/). The data are accessible through the ADNI policies rules.

## Ethics statement

The studies involving humans were approved by Montreal Neurological Institute PET working committee and the Douglas Mental Health University Institute Research Ethics Board. The studies were conducted in accordance with the local legislation and institutional requirements. The participants provided their written informed consent to participate in this study.

## Author contributions

CB: formal analysis, investigation, methodology, original draft preparation, reviewing, and editing. ZC: investigation, methodology, reviewing, and editing. GB: data collection, investigation, reviewing, and editing. JS, NR, CT, and FL: data collection, reviewing, and editing. PR-N, J-PS, and HR: conceptualization, investigation, reviewing, and editing. HB: conceptualization, investigation, methodology, reviewing, and editing. All authors contributed to the article and approved the submitted version.

## Footnotes

^1^ https://triad.tnl-mcgill.com/
